# A Pan-Cancer Analysis of Predictive Methylation Signatures of Response to Cancer Immunotherapy

**DOI:** 10.3389/fimmu.2021.796647

**Published:** 2021-12-09

**Authors:** Bingxiang Xu, Mingjie Lu, Linlin Yan, Minghui Ge, Yong Ren, Ru Wang, Yongqian Shu, Lin Hou, Hao Guo

**Affiliations:** ^1^ School of Kinesiology, Shanghai University of Sport, Shanghai, China; ^2^ State Key Laboratory of Translational Medicine and Innovative Drug Development, Jiangsu Simcere Diagnostics Co., Ltd., Nanjing, China; ^3^ Nanjing Simcere Medical Laboratory Science Co., Ltd., Nanjing, China; ^4^ Shanghai Frontiers Science Research Base of Exercise and Metabolic Health, Shanghai, China; ^5^ Department of Oncology and Cancer Rehabilitation Center, The First Affiliated Hospital of Nanjing Medical University, Nanjing, China; ^6^ Center for Statistical Science, Department of Industrial Engineering, Tsinghua University, Beijing, China

**Keywords:** DNA methylation, predictive modeling, immunotherapy, immune checkpoint inhibitors, support vector machine

## Abstract

Recently, tumor immunotherapy based on immune checkpoint inhibitors (ICI) has been introduced and widely adopted for various tumor types. Nevertheless, tumor immunotherapy has a few drawbacks, including significant uncertainty of outcome, the possibility of severe immune-related adverse events for patients receiving such treatments, and the lack of effective biomarkers to determine the ICI treatments’ responsiveness. DNA methylation profiles were recently identified as an indicator of the tumor immune microenvironment. They serve as a potential hot spot for predicting responses to ICI treatment for their stability and convenience of measurement by liquid biopsy. We demonstrated the possibility of DNA methylation profiles as a predictor for responses to the ICI treatments at the pan-cancer level by analyzing DNA methylation profiles considered responsive and non-responsive to the treatments. An SVM model was built based on this differential analysis in the pan-cancer levels. The performance of the model was then assessed both at the pan-cancer level and in specific tumor types. It was also compared to the existing gene expression profile-based method. DNA methylation profiles were shown to be predictable for the responses to the ICI treatments in the TCGA cases in pan-cancer levels. The proposed SVM model was shown to have high performance in pan-cancer and specific cancer types. This performance was comparable to that of gene expression profile-based one. The combination of the two models had even higher performance, indicating the potential complementarity of the DNA methylation and gene expression profiles in the prediction of ICI treatment responses.

## Introduction

Cancer immunotherapy based on immune checkpoint inhibitors (ICI), such as antibody-mediated interventions targeting cytotoxic T lymphocyte antigen-4 (CTLA-4), programmed death receptor-1 (PD-1) on T lymphocytes, and the principal ligand (PD-L1), has recently caught much attention in the field of cancer therapy for its high efficiency for reversing the tumor-induced immunosuppression and yielding a durable clinical response for a wide range of tumor types. ICIs are now used as single agents or combined with chemotherapies as first or second lines of treatment for about 50 cancer types (1). There are more than 3,000 active clinical trials evaluating ICIs by now, representing about 2/3 of all oncology trials (2).

A major limitation of tumor immunotherapy, especially those based on the ICI, lies in that only a fraction of cancer patients could respond to the therapy (3, 4), and severe immune-related adverse events (irAEs) are frequently seen in some patients undergoing the ICI therapy (5). These adverse events are mainly due to the inhibition of immune checkpoints that reinforce the normal physiological barriers against autoimmunity, leading to various local and systemic autoimmunity (5).

Therefore, the development of the biomarkers evaluating the responsiveness of a patient to the ICI therapy is key for the application of immune therapy in a wider range. Several biomarkers have been proposed, including the tumor mutation burden (TMB) (6), the neoantigens (7, 8), the overexpression of targeting genes such as PD-L1 (9, 10), and the amount and composition of the tumor-infiltrating immune cells (11, 12). However, the predictive powers of these biomarkers are still not applaudable with unwanted tumor type specificity. For example, TMB failed to predict the responsiveness to PD-L1 ICI treatment in non-small-cell lung cancer (NSCLC) (13, 14). The objective response rate to anti-PD-L1 treatment of colorectal cancer (CRC) patients with high microsatellite instability (MSI) values was also only about 40 to 70% (15).

Other than biomarkers mentioned above, DNA methylation could potentially be a rich source of biomarkers for ICI responsiveness (13). Besides its role in tumorigenesis by regulating gene expression (16, 17) and promoting somatic mutations and structural variations (18, 19), DNA methylation profiles have long been recognized as indicators for the status of tumor immune microenvironment as well. For instance, demethylation of transcription start sites (TSSs) of key effector genes, such as Interferon Gamma (IFNG), Granzyme B (GZMB), C-C Motif Chemokine Receptor 7 (CCR7), and Transcription Factor 7 (TCF7), indicates the stimulation of naive CD8^+^ T cells (20). Genome-wide DNA-methylation landscape defines specialization of regulatory T cells (21). Clonal expansion of T cells from naive T cells to effective T cells is associated with distinct DNA methylation landscapes (20). The tumor immune infiltration analysis based on DNA methylation profiles has been successful in a variety of tumor types (22). It has also been pointed out recently in an NSCLC cohort that the global DNA methylation loss is tightly related to the poor outcome of ICI therapy (13).

We first showed the potential of DNA methylation profiles in predicting the responsiveness to ICI therapy and then selected a combination of methylation sites with prediction power. Next, we built a machine learning model to predict immune therapy responsiveness based on the selected methylation features. The model has high prediction accuracy at both pan-cancer level and tumor type specific level. The performance was validated in an independent cohort and is comparable to that of previously reported models based on gene expression profiles. We also showed that the combination of DNA methylation and gene expression profiles overtops models based on single types of biomarkers, indicating the possibility to improve the prediction accuracy by combining different types of biomarkers.

## Materials and Methods

### Datasets

We downloaded the DNA methylation data measured by Illumina Infinium HumanMethylation450 BeadChip (*β* values), together with the gene expression data measured by RNA-seq (read counts), the somatic mutation data (MC3 public version), and the overall survival (OS) information of 32 tumor types from the Cancer Genome Atlas [TCGA, downloaded from the GDC data portal (portal.gdc.cancer.gov) in October 2020]. There were 7,131 cases in total. The name, abbreviations, and number of cases of each tumor type are listed in **Supplementary Table S1**.

The tumor mutation burden (TMB) was calculated as described before with some small modifications (23). In each case, mutations annotated as “Frame Shift Ins”, “Nonsense Mutation”, “Frame Shift Del”, and “Splice Site” were defined as truncation mutations, while those annotated as “Missense Mutation”, “In Frame Del”, “In Frame Ins”, and “Nonstop Mutation” were defined as non-truncation mutations. TMB value was then calculated as


$$TMB=\frac{1}{45}\times (2\times (truncation)+nontruncation).$$

The distribution of calculated TMBs is shown in **Supplementary Figure S1**.

The microsatellite instability (MSI) state for each case was calculated by the MSIpred package as stable or unstable (24).

The cases that simultaneously satisfy the following two criteria are defined as responsive to ICI treatment (positive), and the rest were defined as non-responsive to the treatment (negative). First, the TMB value of the case should be higher than the upper quartile of TMB values in all cases ignoring tumor types. Second, the transforming growth factor β (TGF-β) score [defined in (25) as the weighted average of normalized expression of genes in the TGF-β signaling pathway summarized in (26), taking the regularity direction between genes as weights] should be smaller than the median of the values in all cases ignoring tumor types. It has been shown that both the TMB and the TGF-β score were fine and independent measurements of the responsiveness to ICI treatment (27–31), and the combined measurement has also been successfully used in other pan-cancer studies (32). It is also a practical consideration to define the responsiveness indirectly since there is no large pan-cancer ICI treatment cohort with DNA methylation levels measured for building the models to the best of our knowledge. The number of cases labeled as positive in each tumor type is shown in **Supplementary Figure S2**. It was noticeable that this definition led to severe class imbalance, with 9.86% cases labeled as positive. This issue was solved by the random oversampling scheme in the model-building step described below.

The NSCLC validation cohort data were retrieved from literature (13) and (33). The clinical information was downloaded from the Supplementary Tables in corresponding literature. The DNA methylation profiles were downloaded from the associated datasets (GSE119144 and GSE126043) in Gene Expression Omnibus (GEO). Missing values were imputed as described in the raw pieces of literature (**Supplementary Table S2**).

### Differential Analysis

To perform differential analysis of methylation data, each *β* value was first transformed into M-value by the transformation, $M=\log\left(\frac{\beta }{1-\beta }\right)$ . Then differential analysis was carried out using *limma* package as common practices (34). Probes with absolute *logFC* > 1 and adjusted p-value less than 0.01 were determined as differential methylated probes (DMP).

RNA-seq read counts for each gene were directly inputted into the DESeq2 package for differential expression analysis (35). Genes with *log*_2_ fold change greater than 1 and adjusted p-value less than 0.01 were determined as differentially expressed.

### Predictive Model Building

#### Feature Selection

There are two notable features of DNA methylation data that complicate the analysis. First, the data are high dimensional, with the number of probes (features) amounting to 480K in Illumina Infinium HumanMethylation450 BeadChip. Second, there was also strong collinearity among different probe groups. These two points made most popular *ad hoc* feature selection methods inefficient, which hindered the prediction model building process. Here we describe a feature selection process based on prior knowledge. The basic idea behind this process was that cases in most tumor types could be grouped into two clusters based on the immune infiltration analysis from DNA methylation profiles (22). The two clusters differed in the compositions of immune cells and potentially in the responsiveness to immune therapies. In particular, we applied the analysis pipeline described by Hyunchul et al. to the 32 TCGA tumor types separately (22). In each tumor type, cases were clustered using PAM clustering into two groups, and the group with longer mean survival time was set as cluster 2. The best number of clusters were inspected using a combination of 13 indicators with R package “NbClust,” and an optimal value 2 was observed in 20 out of 32 tumor types (36). For the remaining types, cases were also clearly separated into two groups by visual inspection.

To evaluate the potential relationship between the immune infiltration–based clustering of cases and the responsiveness of the ICI treatment, we selected three indicators of the responsiveness: the overall survival time (OS), the TMB, and the expression level of PD-L1 (CD274) gene. The three indicators were then subjected to appropriate tests for calculating the p-value of differences between the two clusters for each tumor type (log rank test for OS, Mann Whitney U test for TMB, and directly taken from the differential expression results for PD-L1 expression).

For each indicator, differentially methylated probes (DMPs) between the two clusters with significance together with the directions of the differences were extracted in each tumor type. DMPs with the same direction in half or more such tumor types were selected as features. We also added the 1152 probes in the signature used in the immune infiltration analysis with positive *β* value in at least one case (22) to make the final selected feature set.

#### Model Building

**Figure 1:**
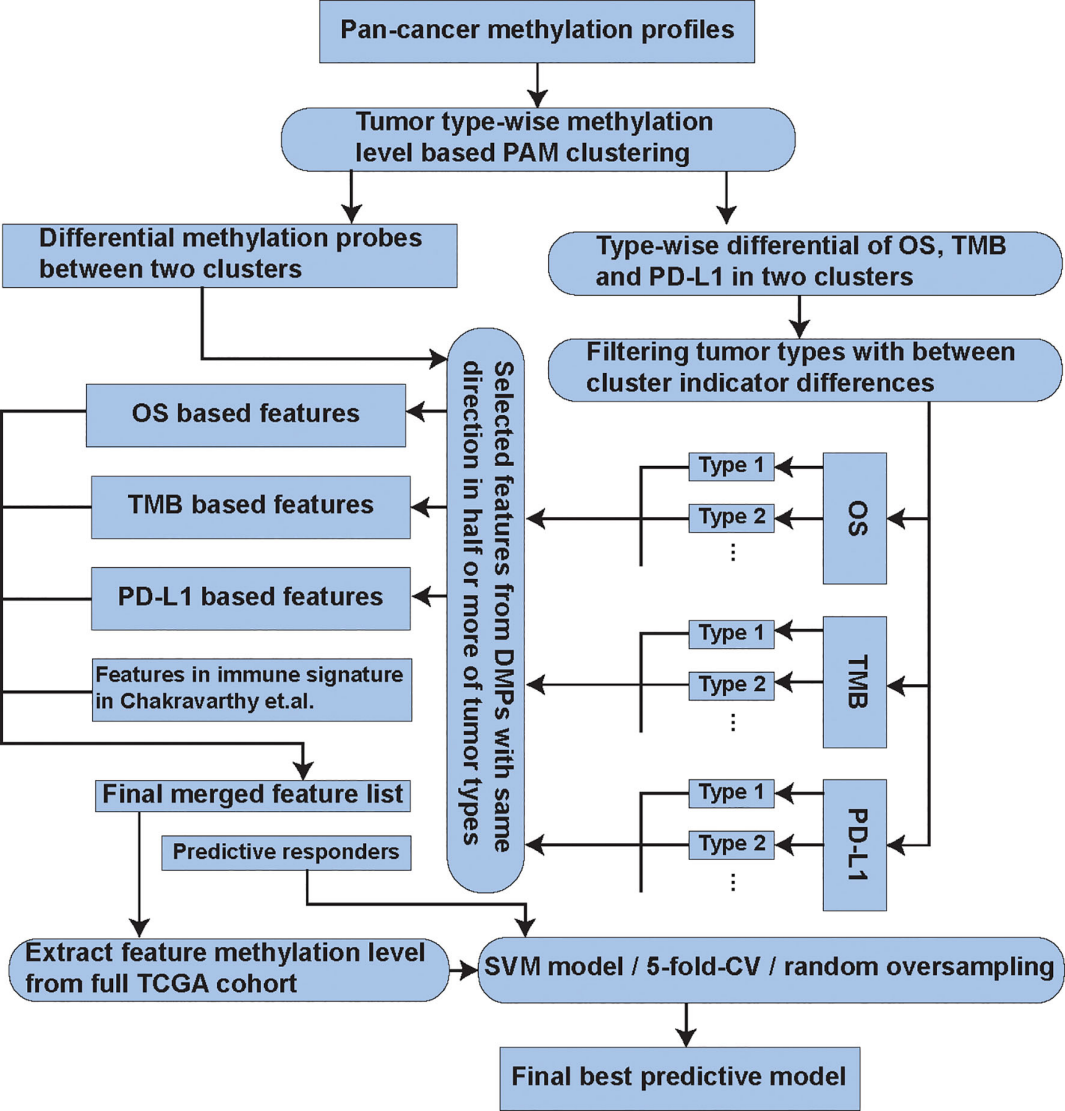
The flow chart describing the process of the feature selection and model building.

We evaluated a series of commonly used machine learning algorithms [logistic regression with *L*_1_ regularization (LR), support vector machine classifier (SVM), random forest (RF), and *k*-nearest neighbor classifier (*k*NN)]. The best parameter combinations for each model were selected by performing a grid search in their spaces. Each parameter combination was evaluated using 5-fold cross-validation. For the SVM model, we used the radial basis function as the measurement of the inner product. The scale parameter of the basis function was taken as the mean variance of all features, and only the multiplier *λ* was tuned for the tune of the scale parameter led to few improvements of the model performance (data not shown). In the model training step, a random oversampling step was added to deal with the severe class imbalance (37). This scheme resampled the positive training cases with replacement to the number of negative ones and then used these balanced training data for model training. The performance of the best models was evaluated by averaging the results of tests on 100 times’ random 80 *vs* 20% train test splits. The *F*_1_ score, Matthews correlation coefficient (MCC), and area under the receiver operating characteristic curve (AUC, if decision scores exist) were calculated as the measurements of the model performance in each test. These measurements were reported to be stable when the classes were severely imbalanced (38). The feature selection and model building processes were illustrated in **Figure 1**.

## Results

### The Responsiveness of Tumor Cases to the Immune Checkpoint Inhibitor Was Highly Related to the Methylation Level of a Small Set of Methylation Sites

This study was focused on predicting the responsiveness of immune checkpoint inhibitor (ICI) at the pan-cancer level. To achieve this goal, the 7,131 TCGA cases from 32 tumor types with both DNA methylation (measured by Illumina Infinium HumanMethylation450 BeadChip, about 480K probes measured in total) and gene expression (measured by RNA-seq) data were downloaded (in October 2020). Cases with high tumor mutation burden (TMB, see the distributions of TMBs in **Supplementary Figure S1**) and low TGF-*β* expression were defined as responsive to ICI (positive) and the others as without responsiveness (negative) as previously suggested (*Methods*, see **Supplementary Figure S2A** for the number of cases marked as positive in each tumor type) (27–29, 32) since large pan-cancer tumor immunotherapy cohort with DNA methylation level measured was lacking. A series of well-known biomarkers, such as the concentration of CD8^+^ T cells, the expression level of PD-1 and CTLA4 genes, were significantly separated between positive and negative cases (all with *p* < 0.01, **Supplementary Figures S2B–D**), elucidated the rationality of our classification. It was reported that the clustering based on immune infiltration analysis using DNA methylation data in most tumor types grouped cases into two clusters, and the clusters were shown to be related to the outcome of immunotherapy (22). Based on this background knowledge, we designed an efficient feature selection scheme to select 1,495 methylation probes from the total 480K candidate methylation probes, which distinguished cases with ICI responsiveness and those without (see *Methods* for detail). In the first step of the feature selection, the analysis was restricted to tumor types with more than 5 cases marked as positive (13 types in total). Tumor cases were clustered into two clusters based on the immune infiltration profiles derived by the DNA methylation profiles. The size of the two clusters for each used tumor type is shown in **Supplementary Table S1**. Then we tested differences between the two clusters in each tumor type for three indicators for the responsiveness of ICI treatment, overall survival (OS), tumor mutation burden (TMB), and the PD-L1 gene expression level. After applying the Benjamini-Hochberg FDR correction scheme, we obtained 6, 7, and 7 tumor types with significant differences of these indicators, respectively (FDR<0.01, see **Supplementary Figures S3A–C** for each indicator **Supplementary Figure S3D** for the overlap of tumor types selected for the three indicators). For each indicator, we selected those methylation probes which differentially methylated with the same direction in at least half of these tumor types with significant differences in the indicators between groups. We got 890, 862, and 534 feature probes for the indicators OS, TMB, and PD-L1 expression, respectively (**Supplementary Figure S3E**). So far, 11 tumor types and more than 85% of positive cases are covered (**Supplementary Figures S2A** and **S3D**). We then merged the feature probes selected for each indicator to obtain 1,495 final probes (the selected features are listed in **Supplementary Table S3**). The 1,152 probes in the signature used in the immune infiltration analysis (22) were added and yielded a final feature set with 2,546 probes.

**Figure 2:**
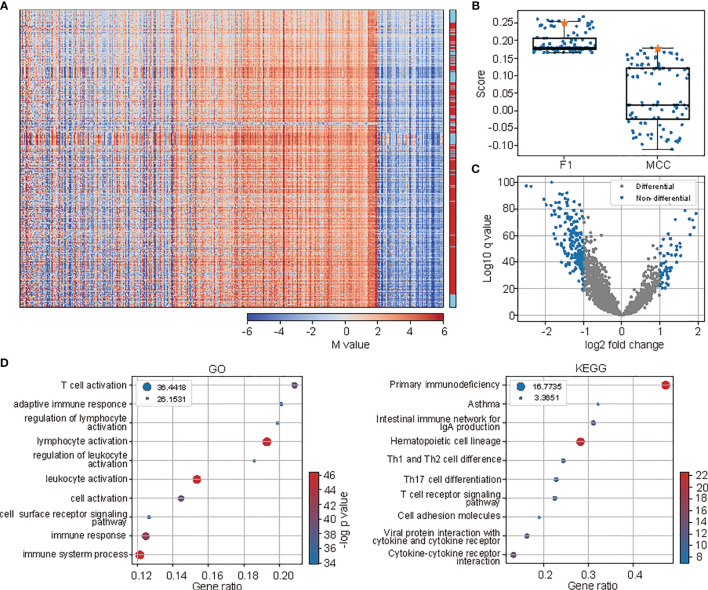
The methylation profiles of selected probes indicated the differences between cases with and without responsiveness to ICI treatment. **(A)** The methylation profiles (M-value) of selected probes of all samples. The samples (rows) and probes (columns) were all rearranged according to hierarchical clustering. Cases with responsiveness to ICI were marked as red at the right panel. **(B)** The comparison of separation between cases with and without responsiveness to ICI based on hierarchical clustering of methylation profiles of selected probes and the randomly selected probes as controls. **(C)** The volcano plot showing the results of differential methylation analysis of selected probes. **(D)** The functional enrichment analysis (left: GO, right: KEGG) of genes where the selected probes were located. The top 10 terms were shown. The size of circles represents the logged FDR values, while colors represent the p-values.

The efficiency of the 2,546 features was revealed by the distinct methylation profiles between cases with ICI responsiveness and those without by visual inspection of the methylation pattern of all cases clustered by the methylation pattern (**Figure 2A**). This pattern was also illustrated by the mutual dependence between the responsiveness of neighboring cases along the dendrogram of the hierarchical clustering (*p* < 10^-52^, *χ* ^2^ test). Moreover, compared to randomly selected, equal-sized control feature sets, clustering based on methylation pattern of selected probes is much better at separating cases with and without ICI responsiveness. In 100 times of such comparisons, the selected features outperformed 92 and 99 random controls when the separation was measured using *F*_1_ score and Matthews correlation coefficient (MCC) score, comparing the responsiveness and cluster labels when clustering the cases into two clusters with the methylation patterns (**Figure 2B**). Also, a large portion of selected probes were under differential methylation between cases with and without responsiveness. When performing the differential methylation analysis among all 32 tumor types, there were 8.76% of selected probes with differential methylation, compared with 5.22 to 7.62% in the 100 randomly selected control features under the threshold of adjusted p-value less than 0.01 (**Figure 2C**). At last, the GO and KEGG enrichment analysis of genes in which the selected probes located showed enrichment of terms related to immune activity and immunotherapy outcome, such as “T cell activation”, “adaptive immune response”, and “regulation of lymphocyte activation” in the GO enrichment analysis, and “primary immunodeficiency” and “Th1 and Th2 cell differentiation” in KEGG enrichment analysis (**Figure 2D**).

### The Responsiveness of Cases to ICI Treatment Could Be Predicted by the Selected Probes

After the prediction potentials for the selected methylation sites for the ICI treatment responsiveness were elucidated, a prediction model was built and tested from various aspects. Here we tested a series of commonly used machine learning models, including the support vector machine (SVM), logistic regression with *L*_1_ regularization (LR), the random forest (RF), and *k*-nearest neighbor classifier (*k*NN). The hyper-parameters of each model were tuned by 5-fold cross-validation. In each model training, the positive cases were oversampled to the size of negative cases to deal with the severe class imbalance (37). The performances of the optimized models were assessed by randomly splitting the raw data into 80% training and 20% test datasets for 100 times. For comparison, we also added a naive predictor based on the clustering results derived from the immune infiltration analysis based on the DNA methylation profiles. In each tumor type, the naive predictor declared the cluster of cases with higher average TMB as positive and the other cluster as negative.

**Figure 3:**
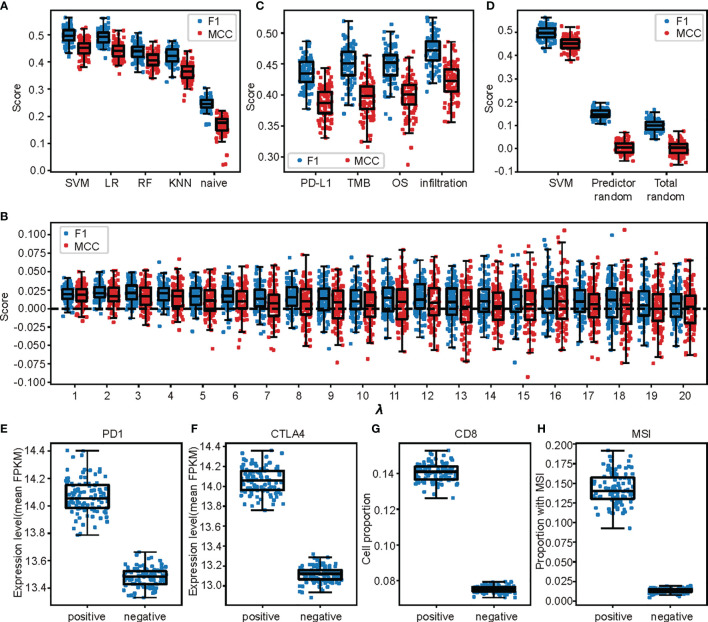
The responsiveness of cases to ICI treatment were predicted by the selected probes. **(A)** The SVM model outperformed the other models when the performances were measured by *F*_1_ score or MCC score. **(B)** The SVM model performances when only probes selected by single indicator involved. **(C)** Differences of the model performances between SVM models trained from the selected probes and random chosen controls among all super-parameter λs searched in the cross-validation step of the model building. In each comparison, samples were randomly split into 80% training and 20% testing set. **(D)** Comparisons between performances of the trained models and those when the respondent (responsiveness) of the samples were randomly shuffled. “Predicted random” meant the predicting performance of SVM models with same setting when the respondent was shuffled. “Total random” meant direct measurement of the similarity of respondents before and after shuffling when the similarity was measured by *F*_1_ score or MCC score. **(E–H)** Differences of biomarkers for ICI treatment responsiveness which is independent with those used in model build for the cases predicted as positive and negative in the 100 random test sets. Each point represented the average value in one test set.

Among the four assessed models, SVM got the highest performance. The fine-tuned SVM model in the 5-fold cross validation (with *λ* = 13) outperformed the other three models (*p* = 0.041, 3.83×10^-28^ and 7.33×10^-32^ for LR, RF, and kNN, respective, paired t test) when the performance was measured using *F*_1_ score, and Matthews correlation coefficient (MCC, *p* = 0.011, 1.30×10^-25^ and 8.11×10^-33^, **Figure 3A**). All the machine learning–based models significantly outperformed the naive predictor in both measurements (all with *p* < 10^-30^, **Figure 3A**).

The prediction power of the model cannot be achieved by randomly selected probes. To show this, we randomly selected methylation probe sets of the same size as the selected probe set for 100 times. The performances were measured by repeatedly training models on 80% randomly chosen samples and tested on the other 20% 100 times along all the regularization parameter *λ* values of the SVM model searched in the cross-validation step (1 to 20). The performances of models based on the selected probes were consistently and significantly higher than those of random controls when the performance was measured by *F*_1_ score, while the same conclusion held under most *λ*s (17 out of 20) when the performance was measured using MCC score (**Figure 3B**).

The probes selected by the four indicators were all important to the model performance. This was shown by retraining and evaluating the SVM model with probes selected by only one indicator. The performances were all significantly decreased compared to the full model no matter measured by the F_1_ score or the MCC score (**Figures 3A, C**, all with p < 10^-4^, paired t tests).

To exclude the possibility that the model performances were due to overfitting, we tested our prediction model with 100 randomly permuted datasets and expected a sharp shrink of model performances in these permutated datasets. We trained and evaluated SVM models with the same super-parameters in each permutated dataset as described above. The performances (measured by both F_1_ and MCC scores) of models in these permutated datasets were only slightly higher than the performance measurements calculated by comparing the responsiveness and its random permutation (**Figure 3D**).

At last, we assessed the separation of a series of well-known biomarkers for ICI responsiveness between groups predicted as positive and negative independent from those used to label the cases. The comparisons were made under the test sets of the 100 randomly trained models in the model evaluation step. First, the PD-1 and CTLA4 gene expression levels were significantly higher in cases predicted as positive than those predicted as negative (both with *p* < 10^-32^, Mann Whitney U test, **Figures 3E, F**). Second, there were also outstandingly more CD8^+^ T cells in cases in the positive cases than negative ones (*p* < 10^-52^, t test, **Figure 3G**). Last, there were higher proportions of cases with microsatellite instability (MSI) (39) in those positive cases than in those negative ones (*p* < 10^-52^, t test, **Figure 3H**).

In conclusion, the fine-tuned SVM model based on the selected probes came up with high performances in prediction of the ICI responsiveness in this pan-cancer cohort. The prediction accuracy is remarkably better than randomly selected probe sets of the same size, and we confirmed the improvement cannot be achieved by overfitting.

### The Prediction Model Based on Methylation Data Was Comparable and Complementary to That Based on Gene Expression Profiles

It was reported previously that the ICI responsiveness could be predicted by the gene expression levels of genes indicating the immune state of the cases in the pan-cancer level (32). Here we compared the performances of the model based on DNA methylation levels with that of models based on gene expression level (32). The gene expression level–based model was built exactly the same as reported before (32), except for taking the advantage of random oversampling to account for the class imbalance. The accuracy of the native built model was consistently higher than that originally reported [the mean and 95% confident interval MCC score under the 100 random test splits were 0.445 (0.371, 0.501) and 0.296(0.287, 0.306), for the native built one and originally reported, respectively] (32).

**Figure 4:**
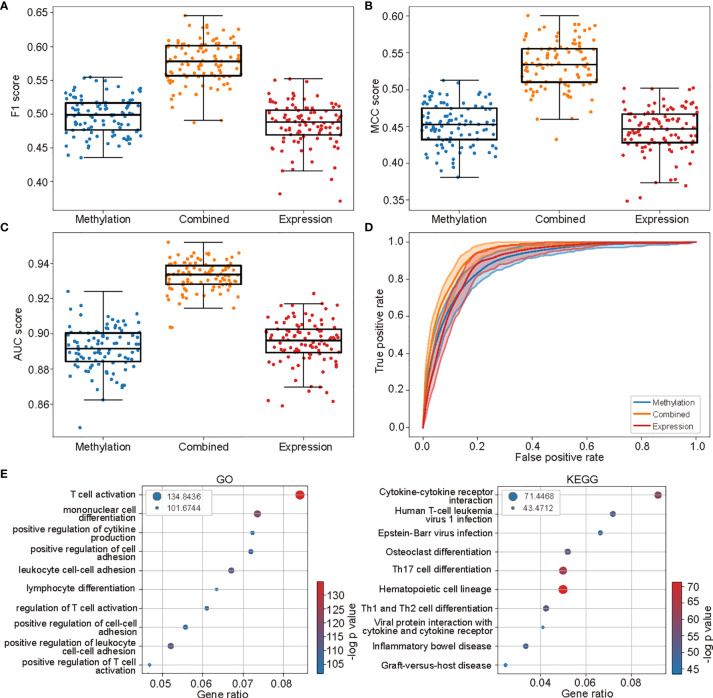
The methylation-based model was comparable and complementary to the gene expression based one. **(A**–**C)** The performances of models based on methylation levels, the expression levels, and the combination of the two along the 100 times random split of the whole cohort into 80% trainings and 20% testing sets. **(D)** The ROC curves of the three models. The shades were 95% confidence intervals along the 100 times splits. **(E)** The top 10 enriched GO (left) and KEGG (right) terms of the genes involved in gene expression–based model.

The performance of the methylation-based model was competitive with that based on gene expression levels. When assessing the performances using 100 times random splits during the model evaluation step, the methylation-based model got better performances when measured by *F*_1_ score (with mean F_1_ score 0.4971 and 0.4844, *p* = 2.82×10^-4^ Wilcoxon signed rank test, **Figure 4A**). The performances were not notably different as measured by MCC score (with mean MCC score 0.4516 and 0.4446, *p* = 0.06, **Figure 4B**), while lower when measured using AUC score (with mean AUC score 0.8914 and 0.8949 *p* = 0.01, **Figure 4C**). The comparability between the performances of two models was also indicated by the closely located ROC curves between the two models (**Figure 4D**).

The genes in which the selected methylation sites located were then compared with those selected in the gene expression–based model. An enormous distinction between the two gene sets was observed. Only 189 out of 2,023 genes selected in the methylation model were found in the genes selected in the expression-based model (2,614 in total). The two gene sets also enriched few common GO and KEGG terms (**Figures 2D** and **4E**). These observations indicated that the prediction power of DNA methylation and gene expression profiles could be complementary, and combining the two profiles would further enhance the prediction power.

To validate our assumption of complementarity of DNA methylation and gene expression profile, we further developed an SVM model based on the combination of the methylation levels of the selected methylation sites and the gene expression levels in the gene expression–based models. The best super-parameter *λ* was selected using 5-fold cross-validation. Performances of the selected model were again assessed in a 100 times random split of the cases into 80% training and 20% testing sets.

The combined model surpassed both the methylation-based and gene expression–based ones under all performance measure [all with *p* < 10^-10^ for measurement *F*_1_, MCC, and AUC scores (**Figures 4A–C**), Wilcoxon signed rank test, with mean scores 0.5773, 0.5340, and 0.9327, respectively]. The high performance of the combined model was also validated by the ROC curves (**Figure 4D**).

These observations have implicated a scheme to enhance the predictive models of ICI responsiveness by assembling the multi-omics data.

### The Methylation-Based Model Accurately Predicts ICI Responsiveness at Specific Tumor Type Level

**Figure 5:**
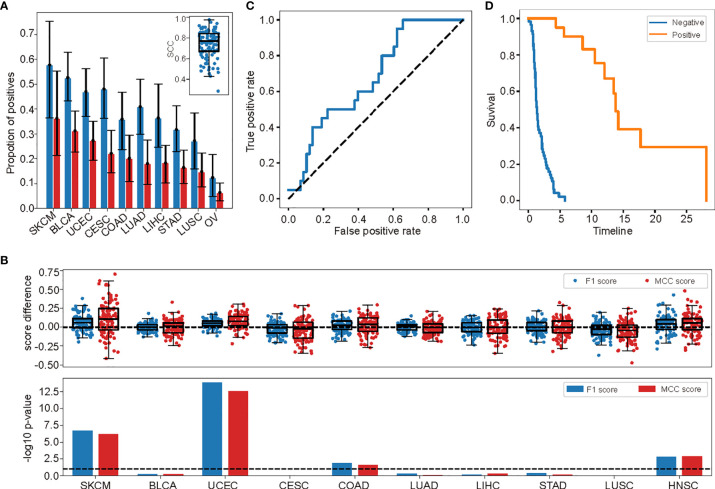
The methylation level–based prediction model was highly performed at specific tumor type level. **(A)** The proportions of cases marked as positive in the 10 investigated tumor types. Bars and error bars indicated the mean and 95% confident intervals among the 100 randomly split test sets. The distribution of the correlation coefficients of these proportions in each test set were shown in the embedded panel. **(B)** The differences of the performance measurements (*F*_1_ score and MCC score) in each tumor type among the 100 randomly split test sets (upper) measuring between model based on the selected probes and randomly selected probes. The significance of these differences (-log10 p-value) were shown in the lower panel. The dashed line marked p=0.1. **(C)** The ROC curve of the independent validation cohort. **(D)** The survival curves of cases predicted as responsive and non-responsive in the validation cohort.

To evaluate the performance of the methylation-based prediction model in each tumor type, we tested the model in tumor types with more than 5%, and 20 cases marked as positive (10 types in total: SKCM, BLCA, UCEC, CESC, COAD, LUAD, LIHC, STAD, LUSC, HNSC, ordered by the proportion of cases marked as position, **Figure 5A**).

One important characteristic of responsiveness to ICI treatment was that the effective rate differed from tumor type to tumor type (40). As in the TCGA dataset, the proportion of cases marked as positive varied largely, from 37% in SKCM to zero in five other types (**Figure 5A** and **Supplementary Table S1**). So, we first investigated that whether the methylation-based model could predict this variation. We calculated the proportion of cases in each tumor type predicted as positive and compared the numbers with the true proportions in the 100 times random test sets. Although due to the high false positive rate, which was the common characteristic of such kind of models (41, 42), the predicted proportion of cases marked as positive were always higher than the ground truths (**Figure 5A**), the two correlated tightly, with average Spearman’s correlation coefficient 0.74 [with 95% confidence interval (0.46,0.93) among the 100 random test sets, **Figure 5A**, embedded panel].

Next, we assessed the performances of the model in each tumor type using *F*_1_ and MCC scores. We compared the performances of the model based on the selected probes in the 100 randomly split train and test datasets with the performances of the models based on randomly selected probes of the same number, under the same hyper-parameter *λ* = 13, which was optimal for the selected probes-based model (**Figure 5B**, upper panel). A paired t test was used to compare the performance between the two types of models. As expected, in 4 out of 10 tumor types, the performance of model based on selected probes was notably higher than that of model based on randomly selected probes (with significant level *p* < 0.1, **Figure 5B**, lower panel). These tumor types included SKCM and HNSC, which were commonly admitted as tightly related to the immune checkpoint evasion and may benefit from the ICI treatment (**Figure 5B**, lower panel) (43, 44). On the other hand, only 2 and 3 tumor types were tested with significantly lower *F*_1_ and MCC scores than the random ones (*p* < 0.1) with the paired t test. This result further illustrated the high performance of the pan-cancer prediction model in tumor type level.

At last, we tested the performance of the methylation-based prediction model in an independent validation cohort. The cohort was taken from two newly published research on non-small-cell lung carcinoma (NSCLC) patients accepting anti-PD-1/PD-L1 treatments with clinical responses measured (13, 33). There were 60 and 18 cases included in the two datasets, with 14 and 6 being identified as responsive to the treatments, respectively. It was worth noticing that this cohort was not included in the TCGA cohort used for model building, and mutation burden was tested as a poor predictor for treatment responsiveness (13). This was the only publically available tumor immunotherapy cohort with DNA methylation levels measured to the best of our knowledge. The methylation levels in this cohort were measured using Illumina Infinium HumanMethylation850 BeadChip. We extracted the probes in the feature set existing in this chip and retrained the model in TCGA data. No significant drop of the performance of the model was observed (with average *F*_1_ and MCC score 0.4949 and 0.4503, with standard deviation 0.0288 and 0.0308). After applying the retrained model in this cohort, it got *F*_1_ = 0.4255, *MCC* = 0.1899, and *AUC* = 0.6742. The performance was also indicated in the ROC curve (**Figure 5C**). The progression-free survival time (PFS) was also significantly prolonged for cases predicted as positive compared with the negative ones (**Figure 5D**), though the p-value (p=0.06, log rank test) was not so significant due to the limited positive cases (only 20 cases predicted as positives). The performance of the model in this totally independent cohort demonstrated its efficiency at both pan-cancer level and for specific tumor type. It also indicated that the information the model caught was indeed the responsiveness itself other than its indicators such as TMB since the model retained its performance when TMB was not predictable to the responsiveness (13).

## Discussion

In this work, we first proposed the potential of the DNA methylation profiles to predict case responsiveness to the immunotherapy using the immune checkpoint inhibitors. Then we designed a feature selection scheme to extract the methylation sites with the prediction power based on the commonly used Illumina Infinium HumanMethylation450 BeadChip measurements of the methylation levels in the pan-cancer level on 32 types of TCGA data. Next, we built a machine learning prediction model for the responsiveness using the methylation levels of these selected sites. The performance of this model was shown both at pan-cancer level and for specific tumor types. The model performance was also compared with that of the existing pan-cancer model based on the gene expression profiles and proved to be comparable and complementary to that model. The combination of the two models was shown to perform better than the single ones. At last, the performance of the model was further shown using a cohort of NSCLC patients. Neither the patients nor the tumor type was involved in the model-building process.

The uncertainty of the outcome and possibility of severe immune-related adverse events were the major issues for the immunotherapy based on the ICIs (3–5). There has been a large number of biomarkers for the prediction of the responsiveness both at genome level and at transcriptome level, such as the tumor mutation burden (6), the microsatellite instability (39), the neoantigens (7, 8), the PD-L1 expression (9, 10), and the tumor immune microenvironments based on the gene expression profiles (11, 12). But the discussion of such biomarkers based on epigenetic signals were far less discussed. The tight relationship between the DNA methylation profiles and the responsiveness to the ICI treatment was only recently shown in separated tumor types (13, 14). Although the close correlation between the DNA methylation profile and the tumor immune microenvironments in the pan-cancer level has been introduced recently (22), there are no direct, systematic discussion of the prediction power to the cases’ responsiveness at this level to the best of our knowledge. Our conclusion of the high performance of the methylation level–based model both at the pan-cancer level and for specific tumor types further illustrated the close relationship between the DNA methylation profiles and the tumor immunotherapy. It also directly offered a framework for outcome prediction of cases that received the ICI treatment. Moreover, it shows the important role of the epigenetic markers in the tumor immunotherapy. On one hand, we should also acknowledge that despite the model performance was applaudable, it still got a high false positive rate, just like other pan-cancer models did (32). This major issue would be improved by the ensemble of multiple types of biomarkers, and we also showed the power of this kind of ensemble by integrating the methylation level and gene expression profiles. On the other hand, other frameworks such as the anomaly detection may help in tumor types with small proportions of cases responding to ICI treatment. The integration of these supervised and unsupervised frameworks may further improve the model performance. Finally, the performance of the model would certainly be improved with the appearance of large tumor immunotherapy cohorts with direct measurements of the responsiveness to the treatments.

The models involved in these studies were all commonly used ones with limited simplicity. We admitted that the model performance would be further improved if more complicated models such as deep neural networks were applied. We did not apply the deep neural networks because the main goal of this study was to deduce the feasibility of the DNA methylation profiles in the prediction of responsiveness of patients in ICI treatments and to introduce the model-building framework. The SVM model, whose performance was high, comparable with and complementary to that of the gene expression–based ones, was suitable enough for these goals. The more powerful models will be discussed in follow-up studies when more high-quality immunotherapy cohorts are available.

We should also acknowledge that the indirect definition of the responsiveness to the ICI treatments introduced irremediable bias of the model. This, together with limited samples in the NSCLC cohort, led to the degradation of the model performance. Unfortunately, there were few currently available tumor immunotherapy cohorts with responsiveness annotated in the pan-cancer level. It is unpractical to build such models with directly defined responsiveness now.

Compared with the state-of-the-art biomarkers, the epigenetic markers such as DNA methylation profiles, histone modifications, chromatin structure, accessibility, and the nucleosome positioning come up with a lot of advantages, such as the low patient invasiveness. For many of epigenetic markers can be measured in liquid biopsies and body fluids (45), contain rich information of life habits and conditions of patients (46), and reveal the origin and evolution of a given disease they carried (47). Their close correlation with the tumor immune microenvironment and importance in the tumor immunotherapy have received more and more attention recently. A series of epigenetic biomarkers for immunocompetent phenotypes have also been established (48). The thorough study of the roles of these markers will certainly mark a new dawn in the tumor immunotherapy.

In conclusion, the DNA methylation profiles were predictable to the responsiveness to the ICI treatments. The built SVM model were well-performed both at pan-cancer level and for specific tumor types. The performance of our model was comparable with and complementary to that of the gene expression–based model.

## Data Availability Statement

The TCGA datasets analyzed during the current study are available in the GDC portal, https://portal.gdc.cancer.gov/. The DNA methylation profiles in the NSCLC cohort was downloaded from Gene Expression Omnibus (GEO) database under the accession number GSE119144 and GSE126043.

## Author Contributions

Study concept and design: HG, LH, and YS. Acquisition, analysis, or interpretation of data: BX, ML, LY, MG, and YR. Drafting of the manuscript: BX, RW, HG, and LH. Study supervision: HG and LH. All authors contributed to the article and approved the submitted version.

## Funding

This work was supported by grants from the Collaborative Innovation Major Project of Zhengzhou (Grant No. 20XTZX08017); the China’s National Key R&D Program (Grant Nos. 2018ZX10305-409-006, 2018ZX10305-409-005); the UK-China Collaboration Fund to tackle AMR (Innovate UK (TS/S00887X/1) and Ministry of Science and Technology (Grant No. 2018YFE0102100); National Natural Science Foundation of China (Grant No. 81702966).

## Conflict of Interest

Authors BX, LY, MG, YR and HG were employed by company Jiangsu Simcere Diagnostics Co., Ltd. and Nanjing Simcere Medical Laboratory Science Co., Ltd.

The remaining authors declare that the research was conducted in the absence of any commercial or financial relationships that could be construed as a potential conflict of interest.

## Publisher’s Note

All claims expressed in this article are solely those of the authors and do not necessarily represent those of their affiliated organizations, or those of the publisher, the editors and the reviewers. Any product that may be evaluated in this article, or claim that may be made by its manufacturer, is not guaranteed or endorsed by the publisher.

## Supplementary Material

Supplementary Figure S1 The tumor mutation burden (TMB, log transformed) calculated for each case involved in this study. The map between abbreviations and associated tumor types could be found in **Table S1**.

Supplementary Figure S2 The rationality of defining the ICI treatment responsiveness using TMB and TGF-*β* indirectly. **(A)** The number of cases defined as positive and negative in each tumor type. **(B**–**D)** The differences of biomarkers independent with those in the definition of responsiveness between cases defined as positive and negative. In each condition, 100 randomly cases were plotted as points.

Supplementary Figure S3 Differential analysis of the three indicators to the responsiveness of ICI treatment. **(A)** The survival curves of cases in the two clusters of the tumor types with significant differences in the overall survival times. **(B)** The distributions of TMBs of cases in the two clusters of the tumor types with significant differences in the TMB values. **(C)** The volcano plot showing the expression differences of CD274 gene between the two clusters in all tumor types. Those tumor types with significant differences were marked as red. **(D)** The numbers of tumor types detected as significant in each indicator and their overlap. **(E)** The number of features extracted for each indicator and their overlaps.

Supplementary Table S1 The abbreviation, names, number of cases, number of cases in each cluster of immune infiltration–based clustering, and number of cases predicted as positive or negative of tumor types involved in this study. The cluster in each tumor type with longer average overall survival time was defined as cluster 2.

Supplementary Table S2 The clinical information and predicted ICI treatment responsiveness of cases in the NSCLC cohort.

Supplementary Table S3 The features selected by each indicator.

## Abbreviations

ICI, immune checkpoint inhibitors; CTLA-4, cytotoxic T lymphocyte antigen-4; PD-1, programmed death receptor-1; PD-L1, the principal ligand; irAEs, immune-related adverse events; TMB, tumor mutation burden; NSCLC, non-small-cell lung cancer; CRC, colorectal cancer; MSI, microsatellite instability; TSSs, transcription start sites; IFNG, Interferon Gamma; GZMB, Granzyme B; CCR7, C-C Motif Chemokine Receptor 7; TCF7, Transcription Factor 7; OS, overall survival; TCGA, the Cancer Genome Atlas; TGF-β, transforming growth factor β; GEO, Gene Expression Omnibus; DMP, differential methylated probes; MCC, Matthews correlation coefficient; AUC, area under the receiver operating characteristic curve.
