# Supplementary figures and images for: A Pan-Cancer Analysis of Predictive Methylation Signatures of Response to Cancer Immunotherapy
Source: Front Immunol. 2021 Dec 9;12:796647. doi: 10.3389/fimmu.2021.796647 (PMC8695566; doi:10.3389/fimmu.2021.796647)

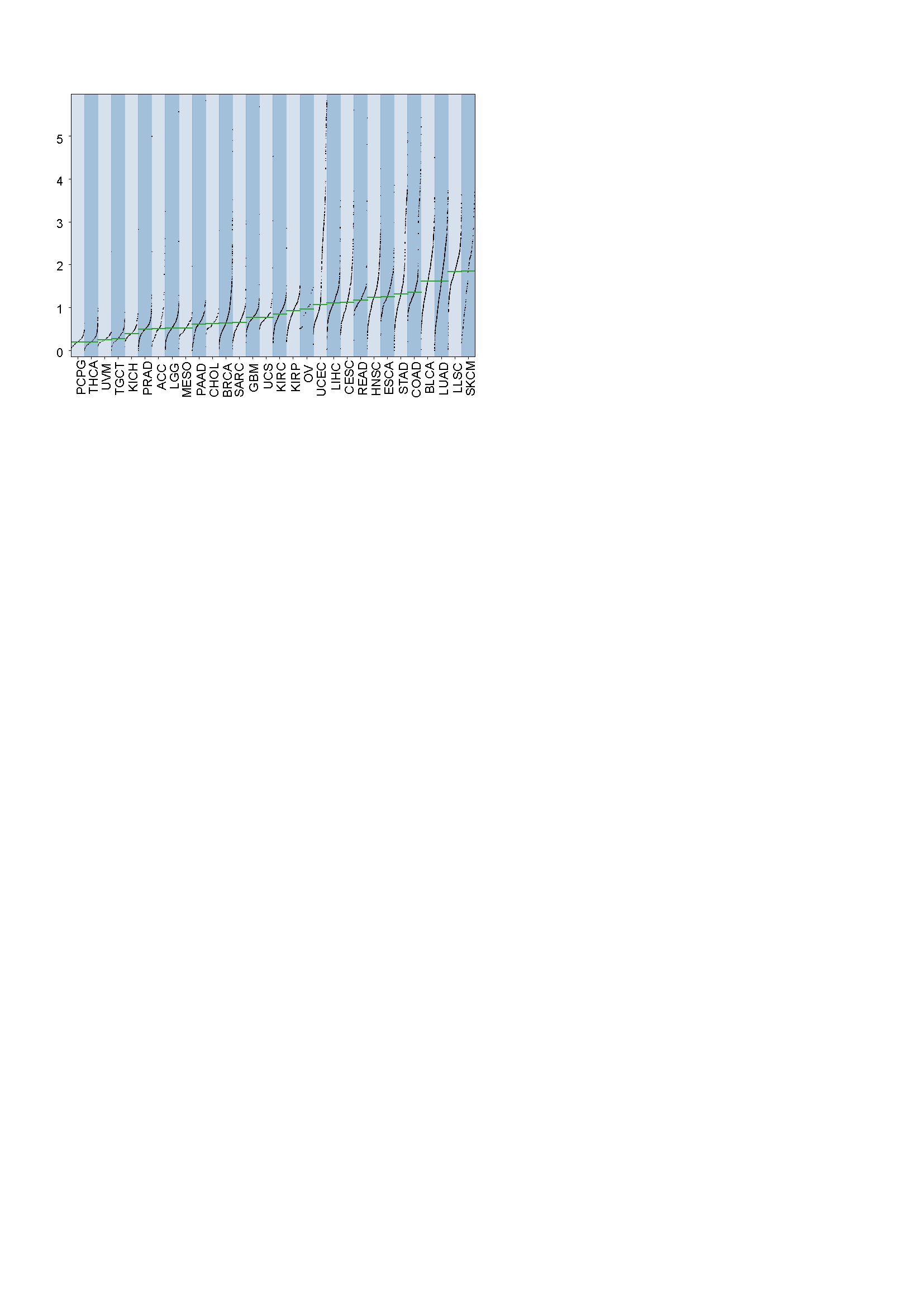

Supplement: Supplementary Figure S1 — The tumor mutation burden (TMB, log transformed) calculated for each case involved in this study. The map between abbreviations and associated tumor types could be found in Table S1 . [file Image_1.tiff]

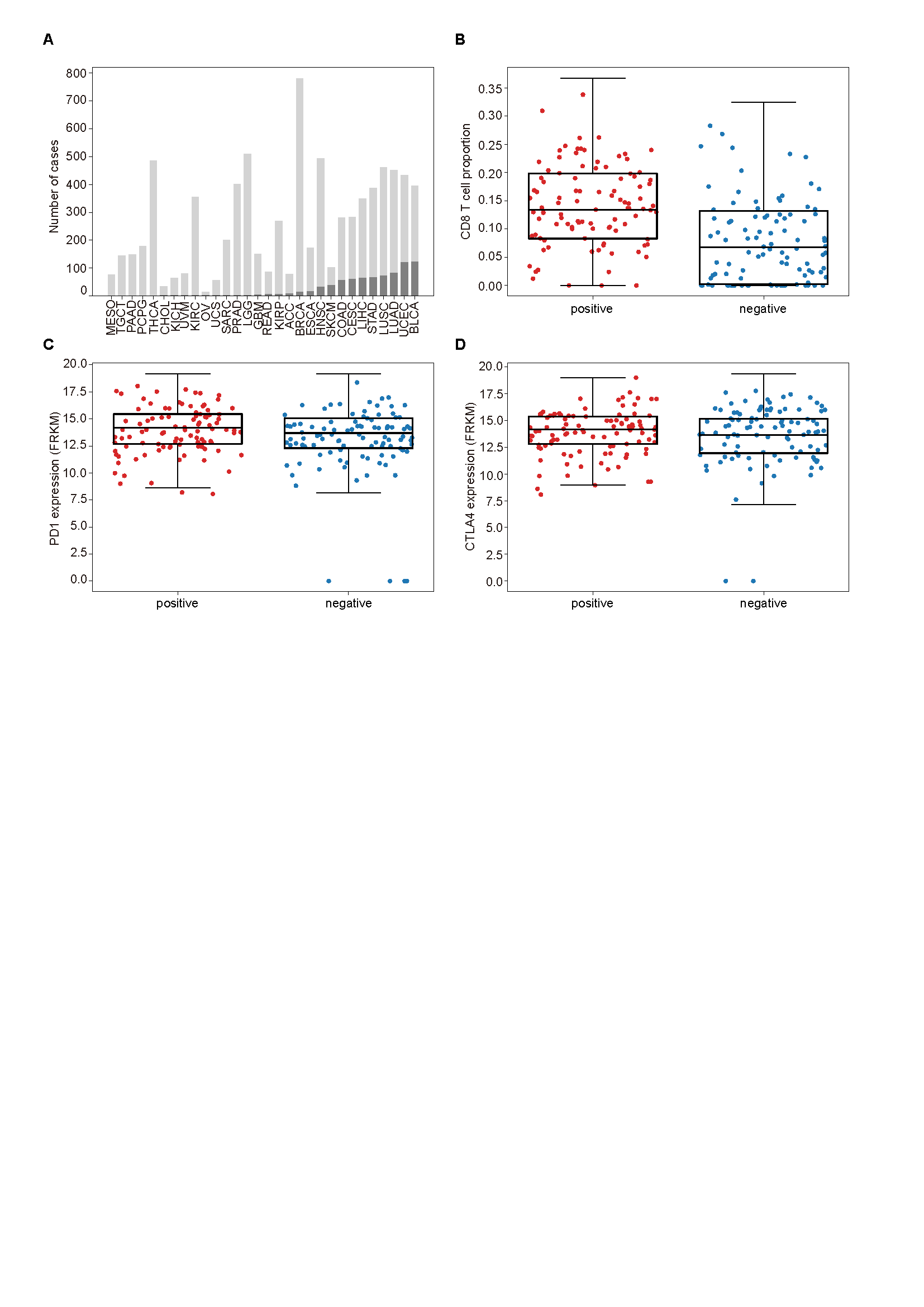

Supplement: Supplementary Figure S2 — The rationality of defining the ICI treatment responsiveness using TMB and TGF-β indirectly. (A) The number of cases defined as positive and negative in each tumor type. (B–D) The differences of biomarkers independent with those in the definition of responsiveness between cases defined as positive and negative. In each condition, 100 randomly cases were plotted as points. [file Image_2.tiff]

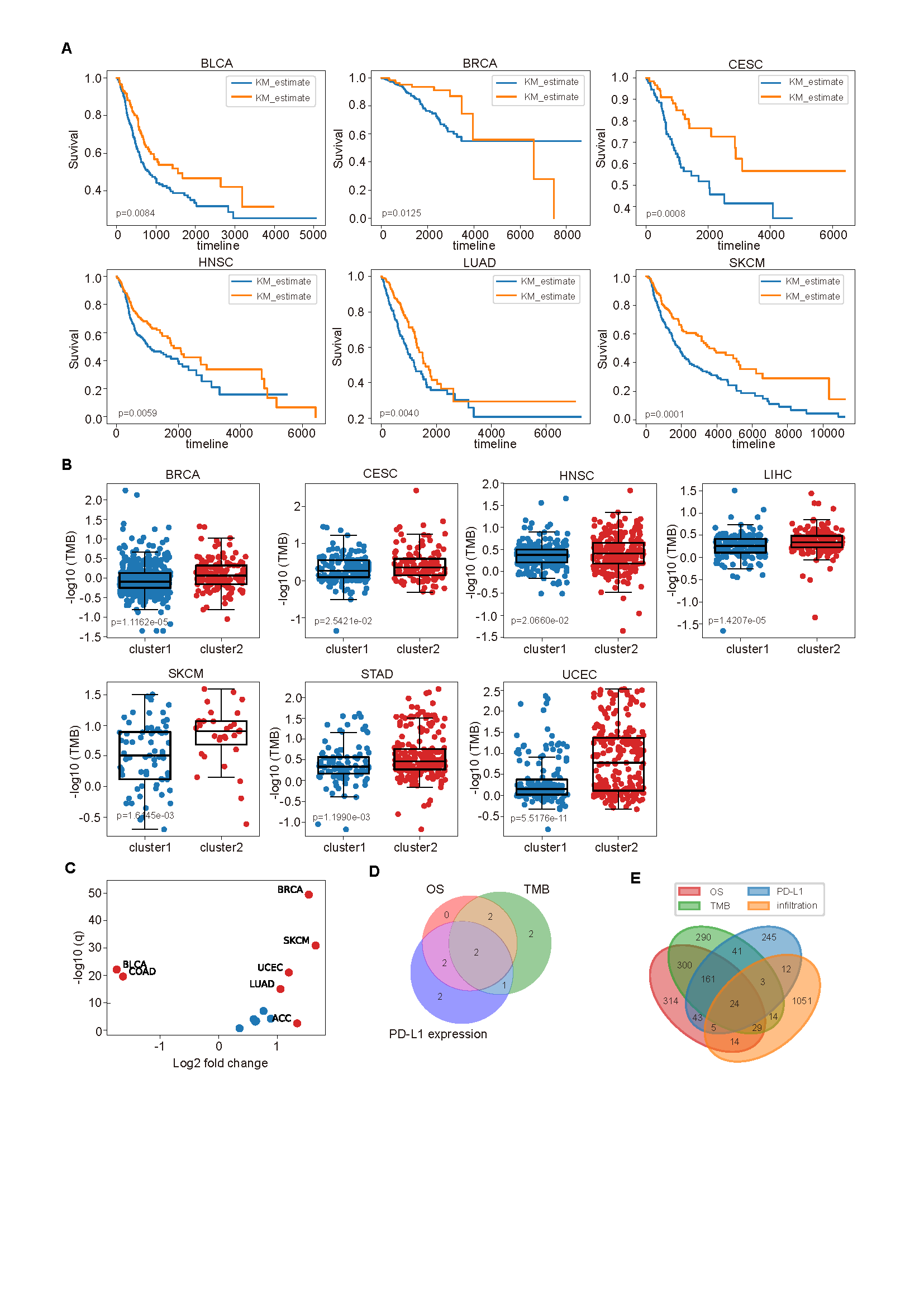

Supplement: Supplementary Figure S3 — Differential analysis of the three indicators to the responsiveness of ICI treatment. (A) The survival curves of cases in the two clusters of the tumor types with significant differences in the overall survival times. (B) The distributions of TMBs of cases in the two clusters of the tumor types with significant differences in the TMB values. (C) The volcano plot showing the expression differences of CD274 gene between the two clusters in all tumor types. Those tumor types with significant differences were marked as red. (D) The numbers of tumor types detected as significant in each indicator and their overlap. (E) The number of features extracted for each indicator and their overlaps. [file Image_3.tiff]
